# Associations between change in blood pressure and functional outcome, early events and death: results from the Efficacy of Nitric Oxide in Stroke trial

**DOI:** 10.1097/HJH.0000000000002154

**Published:** 2019-07-01

**Authors:** Else C. Sandset, Jason P. Appleton, Eivind Berge, Hanne Christensen, John Gommans, Kailash Krishnan, George Ntaios, Stephen Phillips, Stuart Pocock, Nikola Sprigg, Lisa J. Woodhouse, Philip M. Bath

**Affiliations:** a Department of Neurology; b Department of Cardiology, Oslo University Hospital, Oslo, Norway; c Stroke Trials Unit, Division of Clinical Neuroscience, University of Nottingham, Nottingham, UK; d Department of Neurology, Bispebjerg Hospital, Copenhagen, Denmark; e Hawke's Bay Hospital, Hastings, New Zealand; f Department of Medicine, University of Thessaly, Greece; g Division of Neurology, Department of Medicine, Dalhousie University, Halifax, Canada; h London School of Hygiene and Tropical Medicine, London, UK

**Keywords:** acute stroke, blood pressure, glyceryl trinitrate

## Abstract

High blood pressure (BP) is associated with a poor outcome after acute stroke. Early reduction in BP may be associated with fewer early adverse events and deaths, and improved functional outcome.

**Methods:**

Analyses used data from the Efficacy of Nitric Oxide in Stroke trial, a multicentre randomized single-masked and outcome-masked trial of glyceryl trinitrate vs. no glyceryl trinitrate in 4011 patients recruited within 48 h of an ischaemic or haemorrhagic stroke and with raised SBP (140–220 mmHg). Change in SBP from baseline to day 1 was categorized as: more than 15% decrease, 15–5% decrease, 5% decrease to 5% increase (no change – reference) and more than 5% increase. The primary outcome was functional outcome (modified Rankin scale) score at 90 days.

**Results:**

Across all patients, both moderate (5–15%) and large (>15%) decreases in SBP were associated with beneficial shifts in the modified Rankin scale relative to patients with no change in BP: adjusted common odds ratio (OR) 0.81 [95% confidence interval (CI) 0.70–0.90] and OR 0.84 (95% CI 0.71–1.00), respectively. A moderate decrease in SBP was also associated with a lower risk of early adverse events, adjusted OR 0.69 (95% CI 0.52–0.90).

**Conclusion:**

Modest decreases in SBP in acute stroke appear to be associated with fewer early events and better long-term functional outcome.

## INTRODUCTION

High blood pressure (BP) in acute stroke is common [1,2], and has been associated with poor short and long-term outcome in epidemiological studies [1]. Yet, randomized clinical trials have failed to show a beneficial effect of lowering BP in the acute phase [3–5].

One concern regarding early BP lowering, especially in ischaemic stroke, is the risk of cerebral hypoperfusion and progression of infarction. Previous studies have reported associations between large fluctuations of BP in acute stroke and early adverse events such as stroke progression and neurological deterioration [6–8].

The Efficacy of Nitric Oxide in Stroke (ENOS) trial found no overall beneficial effects of BP lowering using a transdermal glyceryl trinitrate (GTN) patch in acute stroke [5]. In the prespecified subgroup of patients treated within 6 h of stroke onset, treatment with GTN appeared to reduce the risk of poor outcome, perhaps by increasing collateral circulation [9,10]. Associations between change in SBP within the first day of randomization, and early events at 7 days and, death and functional outcome at 3 months, are assessed in this secondary analysis of ENOS which involves patients recruited into the trial.

## METHODS

The ENOS study design, statistical analysis plan and participants have been described in detail elsewhere [5,11,12]. Briefly, 4011 patients presenting within 48 h of acute ischaemic or haemorrhagic stroke were randomized in single blind design to treatment with a GTN patch or to no GTN patch for 7 days; those who used BP lowering treatments at the time of stroke were also randomized to continue or stop this treatment in a partial factorial design. At baseline, BP was measured three times – at least one of the three systolic measurements had to be in the range 140–220 mmHg before enrolment and the mean of the three was used in analyses. During the treatment period, BP was measured twice, at least 1–2 h after placement of the daily patch (GTN or no GTN). For all measurements, BP was measured by trained personnel using the nonparetic arm, with the patient supine or sitting; an automated and validated BP monitor was provided to each hospital site for use in the trial (OMRON Healthcare Company, Kyoto, Japan) [5].

### Effect parameters

The primary effect parameter was functional outcome at 90 days, measured centrally by telephone, using the 7-level modified Rankin scale (mRS scores range from 0 to 6, with a score of 0 indicating no symptoms, 1 indicating some symptoms, 2–5 indicating increasing levels of disability and dependency and 6 indicating death). Secondary effect parameters were the end-point of early neurological events, this combining early recurrent stroke or neurological deterioration by 7 days; and death from any cause at 90 days. Early recurrent stroke was classified as haemorrhagic, ischaemic or unknown type. Neurological deterioration was defined as a reduction in Scandinavian Stroke Scale (SSS) score of at least five points, or a decrease in consciousness level of at least three points [5].

### Statistical analysis

All data were collected prospectively, and analyses used the intention-to-treat population of ENOS [12]. The greatest drop in BP occurred between admission (day 0) and day 1; the proportional difference between the measurements was used as a measure for change in SBP: ΔSBP = (SBP day 0 − SBP day 1)/SBP day 0. Patients were categorized into groups according to the proportional change in SBP, identifying patients with a large decrease (>15%), moderate decrease (15–5%), no change (5% decrease to 5% increase, reference group) or increase (>5%). Baseline differences among the four groups were compared using Chi-squared test for categorical variables and one-way analysis of variance with *P* values for linear trend for continuous variables.

Functional outcome at 90 days was analysed using ordinal logistic regression and is reported as adjusted common odds ratios (acORs) with 95% confidence intervals (CIs). The end-points of combined early recurrent stroke and neurological deterioration, and all cause death at 90 days, were analysed using binary logistic regression. Analyses was adjusted for: age, sex, premorbid mRS score, history of previous stroke, history of diabetes mellitus, stroke severity (SSS), stroke syndrome (Oxfordshire Community Stroke Project classification), stroke type (ischaemic, haemorrhagic, unknown type, not stroke), SBP at baseline, treatment with alteplase, feeding status and time to randomization. In the analyses including all patients, adjustment also included GTN vs. no GTN. In addition, to control for the effect of trial treatment, analyses were performed separately for patients in the GTN and no GTN groups, including tests for heterogeneity. As a sensitivity analysis we included the mean SBP [(day 0 + day 1)/2] in the models. All analyses were carried out in the subgroups according to baseline BP, time to randomization and stroke subtype. Analyses were performed using SPSS version 21 (Chicago, Illinois, USA) and 2*P* at least 0.05 is considered significant [12].

## RESULTS

BP measurements were available in 3851 patients (97%). Baseline characteristics are presented in Table 1. A reduction in SBP was observed in 2495 patients (65%) and an increase in 1356 patients (35%). Gradients were apparent across the four groups of patients with those who exhibited a more than 15% decrease in BP having a higher baseline BP and less severe strokes than those whose BP increased over the first day (Table 1). A significantly higher proportion of patients showing a more than 15% decrease in BP were randomized to treatment with GTN (*P* < 0.0001). There were more haemorrhagic strokes among patients with no change in BP, and more patients with total anterior stroke syndromes in patients with an increase in BP.

**TABLE 1:** Baseline characteristics according to change in blood pressure groups

	All	>15% Decrease (large)	5–15% Decrease (moderate)	−5–5% Change (reference)	>5% Increase (increase)	*P*
*N*	3851	593	1121	1412	725	
Sex, female (%)		272 (45.9)	477 (42.6)	573 (40.6)	327 (45.1)	0.081
Age (years)		71.0 ± 11.7	69.9 ± 11.8	70.4 ± 12.0	70.7 ± 13.1	0.26
Premorbid mRS > 0 (%)		164 (27.7)	286 (25.2)	344 (24.4)	205 (28.3)	0.17
Time to randomization (h)		26.7 ± 13.2	25.3 ± 13.0	26.3 ± 12.8	25.6 ± 12.3	0.18
Medical history (%)						
Hypertension		387 (65.3)	728 (64.9)	908 (64.3)	483 (66.6)	0.77
Treated hypertension		333 (56.2)	586 (52.3)	745 (52.8)	395 (54.5)	0.40
Previous stroke		107 (18.0)	166 (14.8)	191 (13.5)	110 (15.2)	0.080
Previous TIA						
Ischaemic heart disease		113 (19.1)	197 (17.6)	237 (16.8)	106 (14.6)	0.37
Peripheral artery disease						
AF, current or previous		108 (18.2)	212 (18.9)	269 (19.1)	146 (20.1)	0.84
Diabetes mellitus		109 (18.4)	190 (16.9)	241 (17.1)	129 (17.8)	0.86
Nitrate use before stroke		26 (4.4)	42 (3.7)	32 (4.4)	48 (3.4)	0.60
Haemodynamics						
SBP (mmHg)		173.8 ± 19.2	169.7 ± 18.8	166.0 ± 18.3	160.3 ± 17.5	<0.0001
DBP (mmHg)		92.7 ± 13.3	90.5 ± 12.3	88.9 ± 13.3	86.2 ± 13.0	<0.0001
Heart rate (bpm)		77.5 ± 15.4	77.6 ± 14.7	77.4 ± 14.6	77.4 ± 14.3	0.99
Qualifying event (%)						
Ischaemic stroke		502 (84.7)	958 (85.5)	1155 (81.8)	602 (83.2)	0.030
Haemorrhagic		84 (14.2)	147 (13.1)	247 (17.5)	116 (16)	
Other		0	0	0	1 (0.1)	
Unknown		7 (1.2)	16 (1.4)	10 (0.7)	5 (0.7)	
OCSP classification (%)						
Total anterior		168 (28.3)	313 (27.9)	423 (30.0)	261 (36.0)	0.030
Partial anterior		188 (31.7)	347 (31.0)	447 (31.7)	219 (30.2)	
Lacunar		212 (35.8)	417 (37.2)	494 (35.0)	219 (30.2)	
Posterior		25 (4.2)	44 (3.9)	26 (3.6)	48 (3.4)	
SSS score (/58)		38 (26–46)	37 (26–45)	36 (24–44)	33 (20–42)	<0.0001
Thrombolytic treatment (%)		67 (11.3)	128 (11.4)	142 (10.8)	79 (10.9)	0.89
GTN treatment (%)		397 (66.9)	607 (54.1)	641 (45.4)	269 (37.1)	<0.0001

Data are *n* (%), median (IQR) or mean (SD). *P* values are based on Chi-squared or Wilcoxon test. SSS scores range from 0 (coma with quadriplegia) to 58 (normal, no neurological deficit). AF, atrial fibrillation; GTN, glyceryl trinitrate; IQR, interquartile range; mRS, modified Rankin scale; OCSP, Oxfordshire Community Stroke Project; SSS, Scandinavian Stroke Scale; TIA, transient ischaemic attack.

**FIGURE 1:**
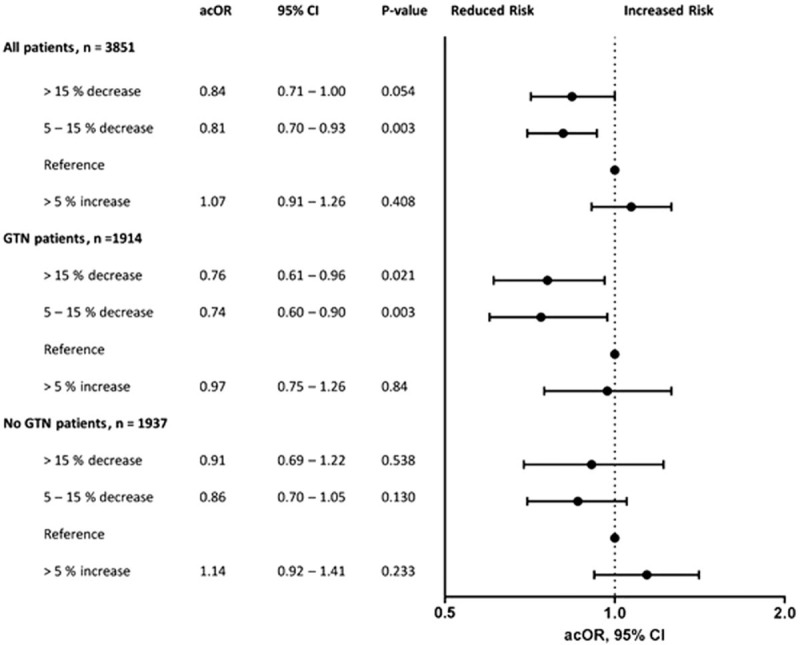
Associations between change in blood pressure and functional outcome at 90 days. acOR, adjusted common odds ratio; CI, confidence interval; GTN, glyceryl trinitrate. Analysed using ordinal logistic regression. ^∗^Adjusted for age, sex, premorbid modified Rankin scale, history of previous stroke, history of diabetes, severity, stroke syndrome (total anterior circulation), stroke type (ischaemic, haemorrhagic, not stroke), SBP, alteplase, feeding status and time to randomization. In the analysis including all patients, we also adjusted for trial treatment.

The association between relative change in SBP and functional outcome is shown in Fig. 1. A moderate reduction in SBP of 5–15% was associated with a beneficial shift in the mRS (OR 0.81, 95% CI 0.70–0.93). Similar results were seen in patients with a more than 15% decrease in BP (OR 0.84, 95% CI 0.71–1.00). There was no association between increase in BP and functional outcome. When assessed by treatment group, the results did not differ between the GTN and no-GTN groups (*P* for interaction = 0.59). Similar results were seen when analysing by absolute rather than relative change (data not shown).

Table 2 shows associations between change in BP and early clinical and neurological events 7 days and death at 90 days. A moderate reduction in BP was associated with fewer early clinical and neurological events (OR 0.69, 5% CI 0.52–0.90), whereas an increase in BP was significantly associated with death at 90 days (OR 1.51, 95% CI 1.14–2.00; *P* = 0.005). Including mean SBP (SBP day 0 + SBP day 1)/2 did not alter the results.

**TABLE 2:** Associations between change in blood pressure and risk of early adverse events (recurrent stroke, clinical and neurological deterioration) at 7 days and death at 90 days

	Recurrent stroke or clinical or neurological deterioration at day 7 Adjusted analysis			Death at 90 days Adjusted analysis		
	OR	95% CI	*P*	OR	95% CI	*P*
All patients						
>15% Decrease	0.96	0.70–1.31	0.026	1.01	0.72–1.42	0.005
5–15% Decrease	0.69	0.52–0.90		0.88	0.66–1.17	
−5–5% Increase	1			1		
>5% Increase	1.04	0.79–1.37		1.51	1.14–2.00	
GTN						
>15% Decrease	1.07	0.71–1.61	0.112	1.15	0.73–1.81	0.192
5–15% Decrease	0.71	0.49–1.05		0.96	0.64–1.44	
−5–5% Increase	1			1		
>5% Increase	1.19	0.78–1.83		1.53	0.98–2.40	
No GTN						
>15% Decrease	0.82	0.49–1.38	0.171	0.89	0.51–1.53	0.021
5–15% Decrease	0.65	0.44–0.96		0.83	0.57–1.24	
−5–5% Increase	1			1		
>5% Increase	0.94	0.65–1.36		1.55	1.07–2.24	

Analysed using binary multivariable logistic regression, adjusted for age, sex, premorbid mRs, history of previous stroke, history of diabetes, severity, stroke syndrome (total anterior circulation), stroke type (ischaemic, haemorrhagic, not stroke), SBP, alteplase, feeding status and time to randomization. In the analysis including all patients, we also adjusted for trial treatment. CI, confidence interval; GTN, glyceryl trinitrate; mRS, modified Rankin scale; OR, odds ratio.

Associations between change in BP and outcome according to baseline SBP are shown in Table 3. In the subgroup of patients with baseline BP 160–179 mmHg, a moderate relative reduction in BP of 5–15% was associated with fewer early neurological events (acOR 0.51, 95% CI 0.32–0.81). In the same subgroup, a relative increase in BP was associated with increased risk of death at 90 days (OR 1.99, 95% CI 1.19–3.32).

**TABLE 3:** Associations between change in blood pressure and early events, functional outcome and death at 90 days, by baseline blood pressure

	Recurrent stroke/Neurological deterioration day 7^a^			mRS day 90^b^			Death at 90 days^a^		
	OR	95% CI	*P*	acOR	95% CI	*P*	OR	95% CI	*P*
SBP < 159 – *n* = 1568									
>15% Decrease	1.22	0.67–2.20	0.249	0.82	0.59–1.13	0.220	0.81	0.42–1.56	0.043
5–15% Decrease	0.65	0.40–1.08		0.73	0.58–0.92	0.009	0.76	0.46–1.25	
−5–5% Increase	1			1			1		
>5% Increase	0.92	0.60–1.40		1.02	0.81–1.28	0.870	1.46	0.97–2.18	
SBP 160–179 – *n* = 1447									
>15% Decrease	0.61	0.34–1.10	0.008	0.80	0.60–1.07	0.128	0.89	0.48–1.67	0.039
5–15% Decrease	0.51	0.32–0.81		0.84	0.67–1.07	0.155	1.17	0.73–1.86	
−5–5% Increase	1			1			1		
>5% Increase	1.13	0.70–1.82		1.24	0.92–1.66	0.156	1.99	1.19–3.32	
SBP 180–199 – *n* = 744									
>15% Decrease	1.01	0.56–1.82	0.95	0.97	0.66–1.42	0.874	1.52	0.75–3.07	0.433
5–15% Decrease	0.90	0.53–1.51		0.87	0.63–1.19	0.380	0.83	0.43–1.59	
−5–5% Increase	1			1			1		
>5% Increase	1.11	0.72–1.71		0.82	0.52–1.2	0.416	1.04	0.45–2.42	
SBP > 200 – *n* = 252									
>15% Decrease	3.12	0.94–10.4	0.126	0.98	0.50–1.90	0.944	0.83	0.27–2.54	0.347
5–15% Decrease	1.02	0.31–3.38		0.71	0.38–1.33	0.287	0.37	0.12–1.23	
−5–5% Increase	1						1		
>5% Increase	2.27	0.60–8.66		0.83	0.35–1.97	0.667	0.81	0.21–3.10	

acOR, adjusted common odds ratio; CI, confidence interval; mRS, modified Rankin scale; OR, odds ratio.

^a^Analysed using binary multivariate logistic regression.

^b^Analysed using ordinal logistic regression, adjusted for age, sex, premorbid mRS, history of previous stroke, history of diabetes, severity, stroke syndrome (total anterior circulation), stroke type (ischaemic, haemorrhagic, not stroke), SBP, alteplase, feeding status and time to randomization.

Table 4 shows associations between change in BP and outcome according to time to randomization. In the group treated very early (<6 h), there was a trend suggesting that a larger decrease was associated with less early neurological events at 7 days (OR 0.24, 95% CI 0.1–0.65). In patients treated after 24 h, an increase in BP was associated with an increased risk of death at 90 days (OR 1.61, 95% CI 1.10–2.37). No difference was seen in the effect of change in BP on either death or functional outcome at 90 days in the other subgroups.

**TABLE 4:** Associations between of change in blood pressure on early events, functional outcome and death at 90 days in subgroups according to time to randomization

	Recurrent stroke/Neurological deterioration day 7^a^			mRS day 90^b^			Death at 90 days^a^		
	OR	95% CI	*P*	acOR	95% CI	*P*	OR	95% CI	*P*
≤6.0 h, *n* = 262									
>15% Decrease	0.20	0.05–0.78	0.015	0.71	0.34–1.51	0.376	0.40	0.06–2.91	0.093
5–15% Decrease	0.24	0.10–0.65		0.80	0.45–1.40	0.431	0.35	0.08–1.55	
−5–5% Increase	1			1			1		
>5% Increase	0.76	0.28–2.05		1.12	0.56–2.26	0.745	2.44	0.68–8.71	
6.1–12.0 h, *n* = 424									
>15% Decrease	0.43	0.15–1.18	0.324	0.82	0.48–1.41	0.476	1.33	0.53–3.36	0.625
5–15% Decrease	0.60	0.26–1.28		0.85	0.55–1.30	0.446	1.44	0.67–3.07	
−5–5% Increase	1			1			1		
>5% Increase	0.67	0.28–1.63		0.63	0.37–1.06	0.082	1.76	0.73–4.22	
12.1–24.0 h, *n* = 1032									
>15% Decrease	1.32	0.76–2.32	0.132	0.71	0.50–1.01	0.055	1.14	0.72–2.63	0.348
5–15% Decrease	0.65	0.39–1.07		0.76	0.58–1.00	0.052	0.85	0.49–1.48	
−5–5% Increase	1			1			1		
>5% Increase	0.95	0.57–1.59		1.01	0.74–1.38	0.943	1.38	0.79–2.40	
>24.1 h, *n* = 1152									
>15% Decrease	1.18	0.76–1.85	0.169	0.93	0.73–1.81	0.554	0.87	0.53–1.43	0.022
5–15% Decrease	0.87	0.50–1.29		0.83	0.68–1.00	0.055	0.87	0.59–1.30	
−5–5% Increase	1			1			1		
>5% Increase	1.40	0.93–2.09		1.17	0.94–1.46	0.155	1.61	1.10–2.37	

acOR, adjusted common odds ratio; CI, confidence interval; mRS, modified Rankin scale; OR, odds ratio.

^a^Analysed using binary multivariate logistic regression.

^b^Analysed using ordinal logistic regression, adjusted for age, sex, premorbid mRS, history of previous stroke, history of diabetes, severity, stroke syndrome (total anterior circulation), stroke type (ischaemic, haemorrhagic, not stroke), SBP, alteplase and feeding status.

In ischaemic stroke a moderate BP decrease predicted improved functional outcome at day 90 (OR 0.76, 95% CI 0.65–0.89, Table 5) and in intracerebral haemorrhage increased BP was associated with increased risk of death at day 90 (OR 2.27, CI 95% 1.11–4.62).

**TABLE 5:** Associations between change in blood pressure on early events, functional outcome and death at 90 days at end of follow-up in subgroups according to stroke subtype

	Recurrent stroke/Neurological deterioration day 7^a^			mRS day 90^b^			Death at 90 days^a^		
	OR	95% CI	*P*	acOR	95% CI	*P*	OR	95% CI	*P*
Ischaemic stroke, *n* = 3218									
>15% Decrease	0.95	0.67–1.35	0.068	0.80	0.66–0.97	0.020	0.87	0.59–1.29	0.017
5–15% Decrease	0.72	0.54–0.97		0.76	0.65–0.89	0.001	0.85	0.62–1.16	
−5–5% Increase	1			1			1		
>5% Increase	1.01	1.00–1.03		1.08	0.90–1.29	0.424	1.43	1.05–1.95	
Haemorrhagic stroke, *n* = 594									
>15% Decrease	0.98	0.47–2.05	0.118	1.14	0.71–1.81	0.589	1.91	0.87–4.21	0.08
5–15% Decrease	0.44	0.21–0.89		1.07	0.74–1.56	0.708	1.11	0.53–2.31	
−5–5% Increase	1			1			1		
>5% Increase	0.71	0.36–1.41		1.17	0.77–1.79	0.456	2.27	1.11–4.62	

acOR, adjusted common odds ratio; CI, confidence interval; mRS, modified Rankin scale; OR, odds ratio.

^a^Analysed using binary multivariate logistic regression.

^b^Analysed using ordinal logistic regression, adjusted for age, sex, premorbid mRS, history of previous stroke, history of diabetes, severity, stroke syndrome (total anterior circulation), SBP, alteplase, feeding status and time to randomization.

## DISCUSSION

In this secondary on-treatment analysis of the ENOS trial, a moderate relative reduction in SBP, independent of treatment assignment, was associated with less early neurological events and better functional outcome, whereas an increase in BP was associated with an increased risk of death.

High SBP at the time of hospital admission has been associated with recurrent stroke, death and poor functional outcome in several populations [1,7,13,14], whereas results regarding change in BP in the acute phase of stroke and outcome are conflicting. A secondary analysis of the Scandinavian Candesartan Acute Stroke Trial (SCAST) found an association between large decreases or increase in BP and early adverse events [6]. However, there was no association between change in BP and long-term functional outcome, as seen in other trials [15]. The results presented here show the opposite, that is, a moderate-to-large relative reduction is associated with better outcome. There may be several reasons for this. First, the patients with a large decrease in BP had lower baseline SBP as compared with the equivalent group in SCAST (173.8 ± 19.2 vs. 182.2 ± 20.2 mmHg). Second, the present analyses used a relative measure of change in BP, which may be more clinically relevant; in contrast, absolute measures of change in BP were used in the SCAST secondary analysis. Finally, the overall achieved BP reduction was greater in ENOS than in SCAST (7/4 vs. 3/1 mmHg on day 1) [3,5].

As reported in other populations, a relative increase in SBP was associated with an increased risk of death at 90 days [15]. Patients in this group had lower BP and more severe strokes at baseline, both known predictors of poor outcome. It is unlikely that there is a causal relationship between increases in BP and risk of death but an increase in BP may be a marker of stroke severity, progression and concurrent disease.

In patients treated very early, a large to moderate relative reduction in SBP was associated with fewer early events. This helps to explain the results of an earlier subgroup analysis where transdermal GTN improved functional outcome, cognition and mood, and reduced the risk of adverse events and death in patients treated within 6 h of symptom onset [9]. Another possible explanation could be the spontaneous fall in BP seen in patients with mild to moderate stroke, who naturally have a better prognosis [16]. Also, regression to the mean may in part have confounded the results since the largest decline in SBP occurred in patients with the highest BP at baseline.

The strength of this study is the large number of patients with acute stroke and high BP with near complete data, including serial standardized BP measurements with a validated BP monitor. The major limitation of the study is that the comparisons were nonrandomized. Further, there is a risk of confounding when combining the GTN and no GTN treatment groups, although the primary outcome was neutral overall. Finally, there is the possibility of chance findings, in part because of multiple testing. As a result, these results should be interpreted with caution.

In conclusion, a moderate-to-large decrease in SBP in acute stroke was safe and associated with fewer early events and better functional outcome at day 90.

## ACKNOWLEDGEMENTS

We thank all the investigators who recruited patients into ENOS, as listed in the main publication.

ENOS was funded by the UK Medical Research Council, and BUPA foundation, and supported by the Stroke Association. E.C.S. is supported by a postdoctoral grant from South Eastern Norway Regional Health Authorities. P.M.B. is Stroke Association Professor of Stroke Medicine and is a NIHR Senior Investigator.

### Conflicts of interest

There are no conflicts of interest.
